# Implementation of EQ-5D-5L as a routine outcome measure in Community Outpatient and Specialized Rehabilitation Services

**DOI:** 10.1186/s41687-021-00369-z

**Published:** 2021-10-12

**Authors:** Katie Churchill, Lisa Warner, Eileen Keogh, Fatima Al Sayah

**Affiliations:** 1 Alberta Health Services, Edmonton, AB Canada; 2 Department of Rehabilitation Medicine, University of Alberta, Edmonton, AB Canada; 3 Department of Clinical Neuroscience, Cummings School of Medicine, University of Calgary, Calgary, AB Canada; 4 Alberta PROMs and EQ-5D Research and Support Unit, School of Public Health, University of Alberta, Edmonton, AB Canada; 5 Allied Health Professional Practice and Education, 10301 Southport Rd SW, Calgary, AB T2W 1S7 Canada

## Abstract

Rehabilitation is a person-centred approach that optimizes functioning to reduce impairments in individuals with illness, injury or disability. Patient-reported outcome measures (PROMs) have a role in rehabilitation to inform clinical practice, enhance patient-centered care, support health services programming, direct performance measurement, and contribute to quality improvement. A Canadian provincial health system implemented a Rehabilitation Model of Care that provides a real-world perspective on the adoption of a standardized PROM, the EuroQol EQ-5D-5L, in the community rehabilitation setting. This article will provide an overview of PROMs implementation in the community rehabilitation context, and discuss key facilitators and challenges to implementation within the 18 early adopter sites and with the spread and scale to 152 urban and rural sites. A change management approach, contextualized local coaching and strong leadership support were facilitators in the initial phases of implementation. Adequate resources and infrastructure from technological platforms for electronic data capture and visualization were assets in addition to clinical teams that had existing strong quality improvement cultures to collect PROMS in existing workflows. Challenges to implementation include the clinical relevance of the PROM, difficulty with contextualization to suit diverse clinical and programmatic teams, and the need for further knowledge sharing activities to build readiness for adoption. The implementation of PROMs in community rehabilitation has added value at the clinical (micro), programmatic (meso) and health system level (macro). Clinically, it has promoted the importance of incorporating the patients’ voice into outcome measurement. At the program level, the cultivation of a data informed learning community was fostered as teams make improvements and use data to inform future program growth or service changes. Finally, at the health system level, data visualization promotes transparency and accountability with performance across the province and the standardized use of the EQ-5D-5L provides a consistent language to promote measurement throughout the health system.

## Background

Rehabilitation is a person-centred approach that optimizes function to reduce impairments in individuals with illness, injury or disability [1]. Patient-reported outcome measures (PROMs) have a role to inform clinical practice, enhance patient-centered care, support health service programming, direct performance measurement, and contribute to quality improvement initiatives [2]. Capturing patient’s perception of their health and health outcomes through the collection of PROMs is one approach used in Canada and around the world to promote patient-centred care [3].

Globally, there is a growing body of literature on the routine use of PROMs within the rehabilitation context [4]; however, consistent and standardized use of PROMs in rehabilitation has not been extensively adopted [5]. In a recent systematic review, Briggs and colleagues report more barriers than facilitators to PROMs use in this setting [5]. Alberta Health Services (AHS) recently implemented a Rehabilitation Model of Care (R-MoC) that provides a real-world perspective on the adoption of a standardized PROM in the community rehabilitation context [6]. PROMs implementation is part of a patient-focused, equitable and data-driven model of care. This paper will provide an overview of PROMs implementation within the R-MoC model in Alberta, and highlight key facilitators and challenges of this process.

## Organizational context

AHS is a provincial health system in Canada whose provision of rehabilitation services in the community supports people by enhancing function, improving quality of life and increasing meaningful productivity [6]. The implementation of the R-MoC in AHS community rehabilitation is in line with the global, national and provincial movement to empower patients in their care, and strives to move the best evidence into practice [3, 6]. The R-MoC has several domains including: wellness; collaborative goal setting; transitions; service options; access and wayfinding and, patient outcomes [6]. The R-MoC outlines the standardized use of outcome measures to capture collaborative goal setting, PROMs, such as health related quality of life (EQ-5D-5L) and a patient reported experience measure. These tools map onto the Health Quality Council of Alberta’s quality matrix elements for health services and includes acceptability, accessibility, effectiveness, and appropriate quality dimensions [7]. These quality dimensions inform future health care implementation efforts, quality improvement, accountability and research [7].

## PROMs implementation

The adoption of the EQ-5D-5L as a PROM in AHS community rehabilitation began in 2017 as part of the R-MoC implementation. The process involved consultations with rehabilitation leaders, clinicians, patients and families, and stakeholders from across Canada and the United Kingdom. It was also informed by completion of a literature review, population needs assessment, current state report, future state review, and a gap analysis to conceptualize the R-MoC. In addition to being the recommended generic PROM by provincial health authorities in Alberta, the EQ-5D-5L was chosen for use in the R-MoC as it could be embedded within workflows to facilitate patient-centred conversations; act as a quality indicator for programs and services; and inform health system planning and decision making.

### Early adopters

To initiate the implementation process, AHS rehabilitation leaders identified teams to be part of the early adopter group (pilot phase) which included the collection of standardized outcome measures. Eighteen teams representing diverse geographical locations and service models stepped forward to begin the process. The implementation of the R-MoC was facilitated through the use of the Institute for Healthcare Improvement’s (IHI) Collaborative Model for Achieving Breakthrough Improvement [8]. The Innovation Learning Collaborative (ILC) process provided the structure to engage teams through learning strategies that included face-to-face learning, independent study, webinars, team driven balanced scorecards and coaching [9]. Provincial rehabilitation consultants provided direct support, engagement and coaching.

### Innovation learning collaboratives

Over the 14 months of the ILC process, the teams came together for three full day face-to-face and one half-day virtual meetings. Agenda items for the meetings included an explanation of the R-MoC, the IHI Model for Improvement, and balanced score cards. Topics included further information on the outcome and experience measures, group programing tips and tricks, resiliency, and opportunities for knowledge sharing. In between meetings, during the action period phases of the ILC, several on-line webinars were offered. Extensive training was conducted with providers to ensure they understood the EQ-5D-5L scores and its interpretation. Providers were encouraged to use the dimension and visual analogue scores to explore patient’s current health status and guide treatment planning. Operational leaders were encouraged to use the index scores for program development and improvement initiatives. In addition to the ILC process, clinicians were provided with training in a behavior change approach to support person-centered care, shared decision making and collaborative goal setting. Learnings from the ILC process included: timely access to data, early support on quality improvement and data management, opportunity to problem solve, and ongoing leadership support. The learnings from the Early Adopter teams (2017–2018) were used to inform the spread and scale of the R-MoC to an additional 152 adult community rehabilitation sites across the province [10].

### PROMs data collection

As part of routine data collection, all community rehabilitation patients who demonstrated cognitive capacity were invited to complete the survey at intake and the end of the episode of care. Examples of the primary reasons for rehabilitation include: musculoskeletal conditions, neurological conditions, respiratory conditions, cardiac conditions, falls, fractures, and stroke.

The intake PROM survey includes the EQ-5D-5L, and questions on age, gender, primary condition, region within the province, and program name. The survey takes approximately 10 min to complete. The PROM surveys are completed via iPad or laptop computer using a secure platform and responses are stored within the AHS firewall. In sites without access to electronic data capture, the survey is paper-based and responses are entered into the secure platform by an administrative member of the rehabilitation team. All patients are assigned a Unique Lifetime Identifier (ULI) to match intake and end of episode of care surveys for further analysis and visualization via Tableau Dashboards.

### Provider feedback

Given the diversity of patients receiving community rehabilitation services, the EQ-5D-5L, a generic PROM, was chosen as the PROM for the R-MoC. Clinician perspective on the EQ-5D-5L implementation was gathered throughout the ILC process by surveys and a focus group. Provider perspective on the use of a generic PROM was mixed. Some providers indicated that administering the PROM provided the opportunity to hear directly from the patient what is important to them, as well as the opportunity to examine program effectiveness. Other providers expressed concern that the EQ-5D-5L lacked specificity and therefore was not applicable to their patients or practice area. For example, the interpretation of “walking about” in the mobility dimension was not relevant for patients who use mobility devices, such as wheelchairs. Consistent with other published literature, teams acknowledged that the addition of a diagnostic specific PROM would add greater value from a clinical perspective [5]. The addition of a diagnostic specific PROM may be considered in the future but currently community rehabilitation clinicians and leaders are only encouraged to supplement their EQ-5D-5L outcome measure collection with diagnostic specific PROMs that reflect the patient population and rehabilitation services they provide.

### Displaying EQ-5D-5L results—Tableau dashboard

**Fig. 1:**
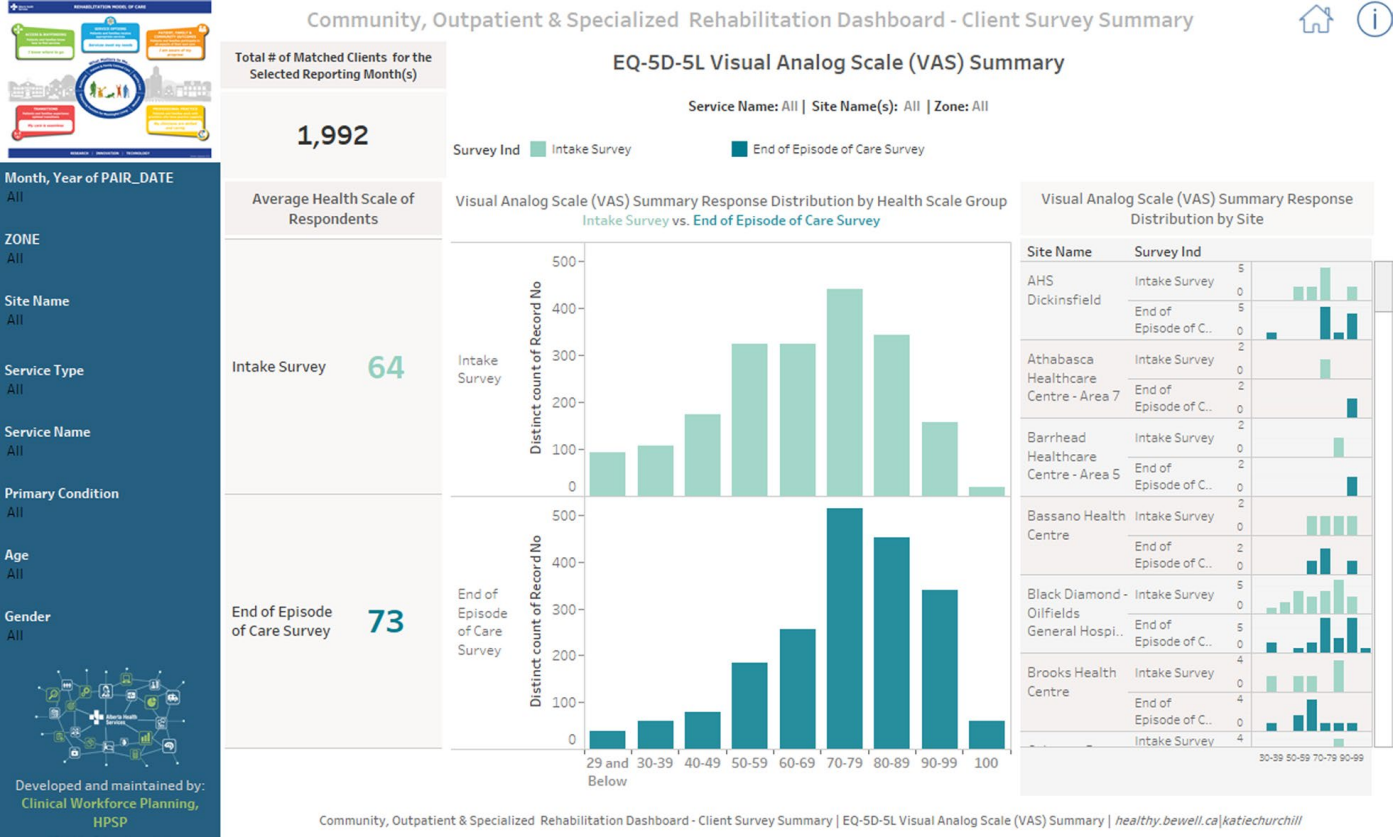
AHS Tableau Dashboard for Community Rehabilitation—Visual Analogue Summary view. In this figure, the EQ VAS score for 1992 patients with complete data at intake and end of episode of care is presented; mean of 64 at intake, and 73 at the end of episode of care. The distribution of scores at both time points is also presented. The summary also showcases (right) the distribution of scores by site

**Fig. 2:**
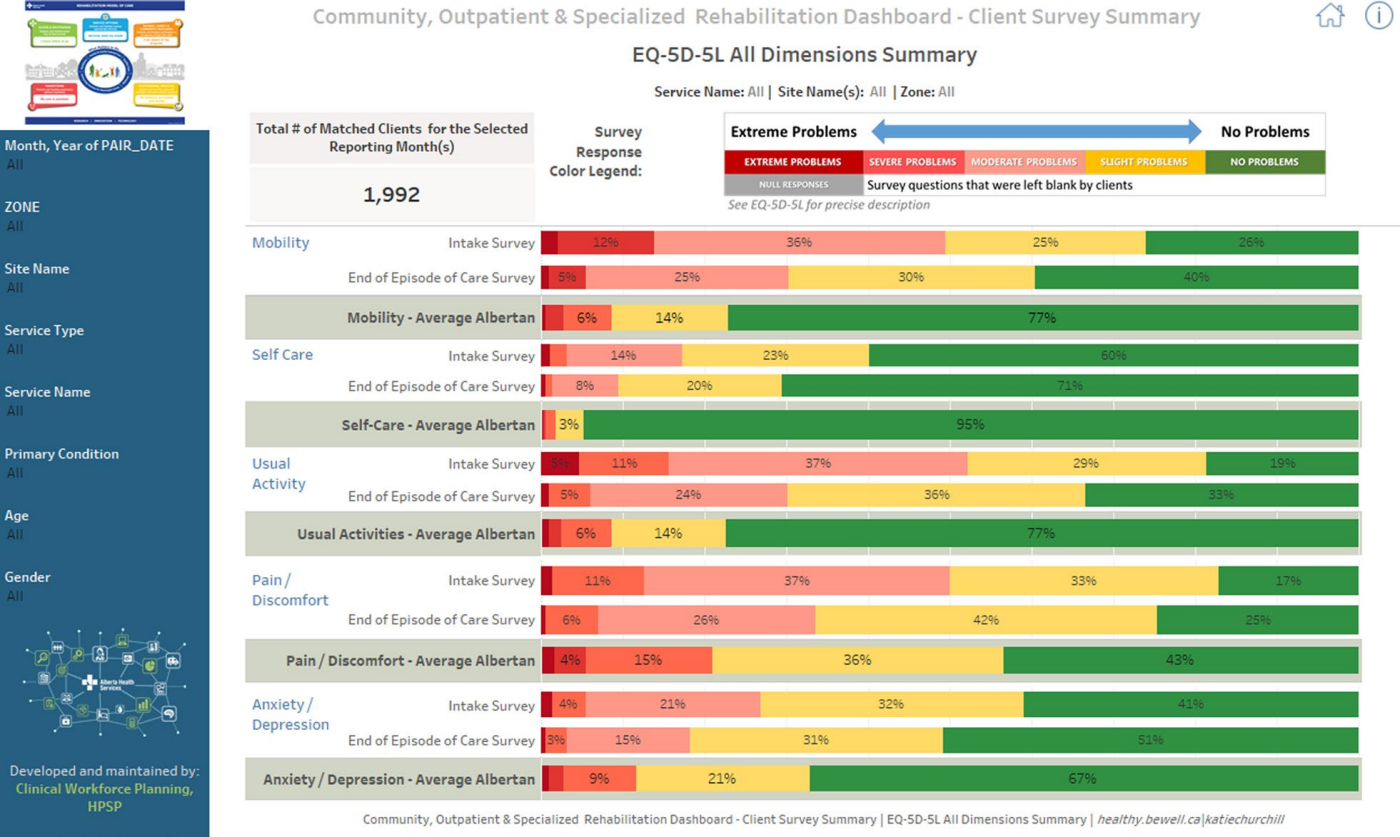
AHS Tableau Dashboard for Community Rehabilitation—EQ-5D-5L Dimensions Summary view. In this figure, the distribution of the EQ-5D-5L levels across the five dimensions for 1992 patients with complete data at intake and end of episode of care is presented, along with the general Alberta population norms data for comparative purposes. A legend for color coding for the levels is also provided for ease of interpretation. Dashboard data is from December 2018–October 2020

The PROM survey data is aggregated and uploaded on a monthly basis to the AHS R-MoC Tableau Dashboard. Tableau is a secure online platform that provides visualization and data analytics. [Figs. 1 and 2]. For data privacy and security purposes, access to the dashboard is restricted to the leaders of the participating sites involved in service improvements. The dashboard can be filtered by zone, service, site, service type, primary condition, age, and gender. Analytics are collected on visits to the R-MoC dashboard and ongoing improvements are made to facilitate data use and promote data informed decision making.

## Discussion

The R-MoC was implemented to cultivate a patient-focused and data-informed service model. Implementing the EQ-5D-5L as a PROM in a provincial context resulted in many learnings, both with the initial pilot implementation of the 18 early adopter teams, as well as the spread and scale to 152 urban and rural sites [11].

### Facilitators

The importance of adequate support and resources has been well established in the literature [5]. The R-MoC success with implementation was facilitated by the infrastructure set up by the ILC process for early adopter teams; contextualized coaching provided by the provincial rehabilitation consultants within the zones; strong operational and management support; and the PROMs infrastructure provided by the technological platforms for electronic data capture and visualization. The characteristics of the teams also had an impact. Teams already collecting outcome measures in their clinical workflows and those with existing quality improvement processes were more successful in adapting their processes to integrate the PROM into data collection. In addition, access to an external centre of excellence, such as the Alberta PROMs and EQ-5D Research and Support Unit (APERSU) at the University of Alberta, was vital as a resource and facilitator to develop buy-in with the operational and clinical teams.

### Challenges

Understanding the challenges of implementation was important so that further refinements to the implementation process can be realized. Due to the diversity of teams, service areas and contexts there could not be a standardized provincial approach. Some teams found it challenging to integrate the survey into their workflows. Additionally, some teams found that the chosen PROM (EQ-5D-5L) did not provide significant clinical or programmatic value in comparison to condition specific PROMs, especially with respect to therapeutic areas or aspects of health not captured by the EQ-5D-5L dimensions. Adjunct education sessions were required to develop knowledge and skills in the PROM tool before teams felt confident to implement it. This observation is consistent with other reports in the current literature [5, 11]. The data collection method also impacted the teams’ readiness to adopt the tool. Paper-based data collection provided an improved opportunity for clinicians to review responses with the patient and incorporate their responses into their treatment planning. The collection of PROMs via iPads made patient responses less accessible to providers thereby creating a barrier for use at the clinical level.

### Micro, meso and macro level applications

The routine collection of PROMs in has the potential to inform decision at various levels within the system. For instance, at the micro (clinical patient management) level, rehabilitation professionals have the opportunity to review EQ-5D-5L responses with the patient and use it to discuss current health status and rehabilitation goals. It supports the clinician to be intentional about including the patient’s voice in their care. It has been acknowledged that PROMs can assist rehabilitation professionals with clinical decision making and collaborative goal setting, and can enhance shared decision-making between patient and provider [5, 12]. Further exploration into PROMS use at the micro level in AHS community rehabilitation is warranted as the implementation moves toward spread and scale. Current data collection systems do not allow for observing changes in PROMs scores over time, which has been reported as a barrier to the usefulness of PROMs by clinicians for decision making, care planning and in practice [13]. As well, the current data collection system does not feedback individual patient results to the clinicians which is a limitation of the technology platform. As AHS moves towards a unified provincial electronic medical record, clinicians may have increased access to individual and programmatic PROMs data, thus addressing some of the potential barriers to use.

At the meso (organization) level, aggregate PROMs data could be used to inform performance measurement through national monitoring and drive quality improvement initiatives, especially when combined with other condition specific outcomes and patient experience measures [12]. Within the R-MoC, we have been using aggregate data to examine the impact of rehabilitation services on patient-reported health status across various community rehabilitation programs and sites, and identify factors associated with improvements and deteriorations in health status [14]. Results indicate that across all community rehabilitation programs, 56% of patients reported significant clinical improvements in their self-reported health status following rehabilitation. This data is also being used to explore the relationship between patient-reported experience and change in PROMs across various community rehabilitation programs.

At the macro (system) level, aggregate PROMs data will be used to compare the performance of rehabilitation teams across sites and health zones in our province. Current data in terms of sample size does not permit yet such comparisons. An overall threshold of 60% achieving an improvement, based on the minimal important difference of the EQ-5D-5L index score, has been established by our PROMs program team. Service specific thresholds are being considered. Additionally, we are planning PROMs data linkage to administrative claims databases that would allow exploring health service utilization and conducting economic analyses.

### The value of PROMs

The implementation of PROMs in community rehabilitation has added value on multiple levels. Clinically, it has promoted the importance of incorporating patients’ voice in outcome measurement. It has also encouraged clinicians to be more intentional about shared decision-making, with the co-construction of goals and using person-centred behavior change strategies to have thoughtful and meaningful conversations with patients. At the program level, an increase in the quality improvement culture of the teams and the development of a learning community had developed in community rehabilitation. Teams are becoming data informed as they make improvements and inform future program or service changes. Tableau dashboards displaying all the outcome measures promote transparency and accountability with performance. Standardized use of the EQ-5D-5L has also provided a consistent language to promote measurement throughout the health system.

## Conclusion

The implementation of a PROM as part of the R-MoC in a provincial health setting has been a large undertaking with many lessons learned. Mitigation strategies to overcome barriers to PROMs use in practice were employed during the implementation phase, such as robust clinician training, leadership support, PROM collection and reporting infrastructure and access to the EQ-5D-5L tool and training, among others. The implementation of the R-MoC, including a PROM as part of routine data collection in community rehabilitation, has increased the quality improvement culture with the clinical teams collecting and analyzing data. It has informed characteristics and health related quality of life of patients accessing community rehabilitation, and will help direct where future efforts need to be focused. Ongoing work with PROMS data at the clinical level will be explored to further inform clinical interventions and care planning.

## Data Availability

The datasets generated and analyzed during the current study are not publicly available but can be made available from the corresponding author upon reasonable request.

## Abbreviations

AHS: Alberta Health Services
APERSU: Alberta PROMs and EQ-5D Research and Support Unit
EQ-5D-5L: EuroQol 5 dimensions, 5 level questionnaire
IHI: Institute for Healthcare Improvement
ILC: Innovation Learning Collaborative
PROMs: Patient Reported Outcome Measure
R-MoC: Rehabilitation Model of Care
ULI: Unique lifetime identifier
